# A Low-Cost, Disposable and Portable Inkjet-Printed Biochip for the Developing World

**DOI:** 10.3390/s20123593

**Published:** 2020-06-25

**Authors:** Kushal Joshi, Vanessa Velasco, Rahim Esfandyarpour

**Affiliations:** 1 Department of Biomedical Engineering, University of California, Irvine, CA 92697, USA; kushalj@uci.edu; 2 Biochemistry Department, Stanford University, Palo Alto, CA 92697, USA; vvelasco@stanford.edu; 3 Department of Electrical Engineering, University of California, Irvine, CA 92697, USA; 4 Henry Samueli School of Engineering, University of California, Irvine, CA 92697, USA

**Keywords:** biochip, electrowetting on dielectric (EWOD), digital microfluidics (DMF), point-of-care (POC) diagnostics, developing world, portable

## Abstract

Electrowetting on dielectric-based digital microfluidic platforms (EWOD-DMF) have a potential to impact point-of-care diagnostics. Conventionally, EWOD-DMF platforms are manufactured in cleanrooms by expert technicians using costly and time consuming micro-nanofabrication processes such as optical lithography, depositions and etching. However, such high-end microfabrication facilities are extremely challenging to establish in resource-poor and low-income countries, due to their high capital investment and operating costs. This makes the fabrication of EWOD-DMF platforms extremely challenging in low-income countries, where such platforms are most needed for many applications such as point-of-care testing applications. To address this challenge, we present a low-cost and simple fabrication procedure for EWOD-DMF electrode arrays, which can be performed anywhere with a commercial office inkjet printer without the need of expensive cleanroom facilities. We demonstrate the utility of our platform to move and mix droplets of different reagents and physiologically conductive buffers, thereby showing its capability to potentially perform a variety of biochemical assays. By combining our low-cost, inkjet-printed EWOD-DMF platform with smartphone imaging technology and a compact control system for droplet manipulation, we also demonstrate a portable and hand-held device which can be programmed to potentially perform a variety of biochemical assays.

## 1. Introduction

The need for automated, high throughput, and reduced cost biochemical analytical techniques has driven the miniaturization of many conventional assays [1,2]. The downsizing of these analytical methods has tremendously benefitted from the low sample volume requirement, improved sensitivity and selectivity of microsensors, reduced turnover times, enabled parallel analyses and batch manufacturing processes. While conventional diagnostic assays usually require milliliters or hundreds of microliters of reagents [1,3], reagent consumptions are lowered by a factor of 10^3^–10^4^ for miniaturized diagnostic assays. Thereby, this has resulted in dramatic savings for repetitive tests that are performed in clinical diagnostic laboratories [1]. In addition to cost savings, miniaturized diagnostic assays with low sample requirements are advantageous in situations where the extraction of a large quantity of patients’ samples could be detrimental to patients’ health. For instance, newborns with iatrogenic anemia have an average total blood volume of 240 mL and can be as low as 60 mL in extremely low birth weight (ELBW) newborns [3]. Conventional blood analysis requires 0.5 to 2.5 mL per test, and several days of testing can lead to blood loss exceeding the circulating blood volume in ELBW newborns [3]. In such cases, miniaturized assays requiring nanoliters to few microliters of blood could be potentially lifesaving.

Additionally, microfluidic systems facilitate the miniaturization of biochemical assays, as they allow for the precise control of fluids at the microscale, which enables highly parallelized experiments with a minimal amount of reagents [4]. Depending on how the fluids are controlled and manipulated, microfluidics devices are divided into two classes: continuous flow and digital (droplet-based) microfluidics [5]. In continuous flow microfluidics, external pumps are usually used to drive fluid flow in the microchannels [5]. On the other hand, digital microfluidics (DMF) involves the manipulation and control of small, discrete liquid droplets within the microliter range or smaller [5]. Unlike continuous microfluidic systems, DMF devices do not need external modules or complicated channel geometries such as pumps or valves to control the fluid flow [6]. Instead, DMF implements electrical forces, to control and move liquid droplets across a surface/duct. With an appropriate DMF design, droplets can be moved, mixed, and split on a plate, facilitating multiple biochemical assays such as protein detection assay [7], fluorogenic apoptotic assay [8] and enzyme-linked immunosorbent assay (ELISA) [9].

**Figure 1:**
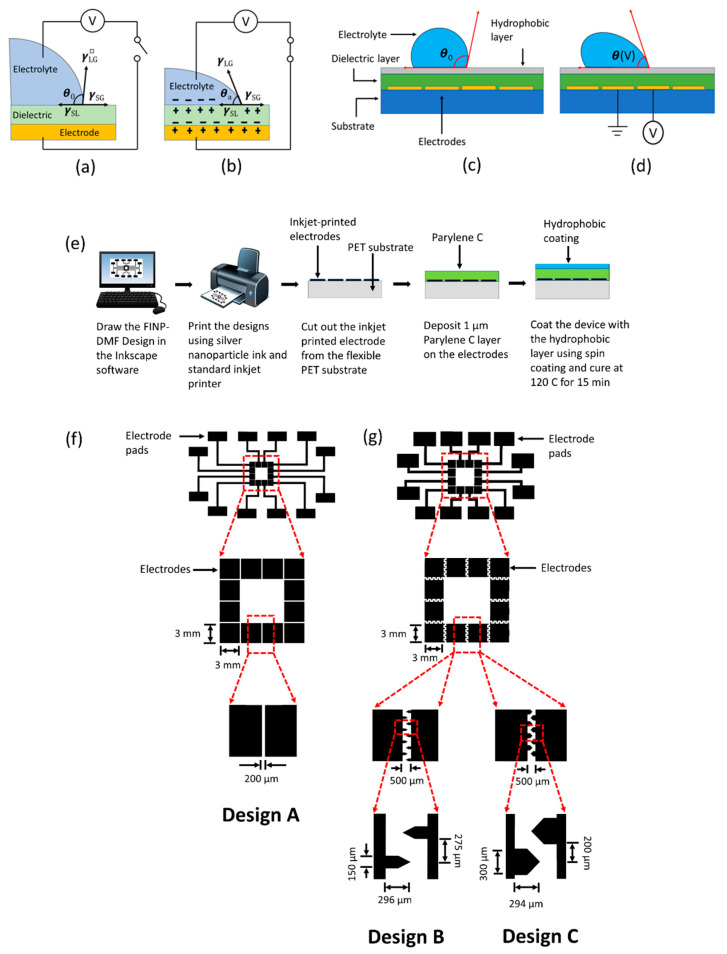
(**a**) A droplet of an electrolyte sitting on an electrowetting on dielectric (EWOD) device. The dielectric layer prevents spontaneous charge accumulation at the surface at zero voltage. (**b**) When a voltage is applied, charges accumulate at the interfaces, which decreases the surface tension of the solid-liquid interface and the contact angle of the liquid. (**c**,**d**) Principle of droplet motion on the EWOD device. When the droplet is located on the boundary between the actuated and non-actuated electrode, the electrowetting force is applied on the contact line located above the actuated electrode, and the capillary force is exerted on the contact line located on the lyophobic region. The resultant force is located towards the actuated electrode, and the droplet moves towards the actuated electrode. (**e**) The fabrication process of the flexible inkjet-nanoparticle-printed digital microfluidic (FINP-DMF) platform. Electrode designs are drawn in the Inkscape software and then printed on a flexible PET substrate using a commercial office printer. The printed devices are then coated with a 1 µm parylene C layer. A hydrophobic layer of Fluoropel PFC-1101V is coated on the top of the device. (**f**) FINP-DMF platform with square electrode configuration (design A). Each electrode is 3 mm × 3 mm, and the gap between two adjacent electrodes is 200 µm. (**g**) FINP-DMF platform with interdigitated electrode configuration (designs B and C). The base size of the electrode is 3 mm × 3 mm. The saw-tooth-like interdigitated features are 150 μm in width for design B and 300 μm for design C.

In particular, electrowetting on dielectric (EWOD), a popular DMF technique, has been used for a variety of applications in biology and medicine [5]. In EWOD, a base plate with an array of discrete electrodes is used to actuate sessile droplets. A dielectric layer is coated on the electrode plate along with a hydrophobic layer on top to increase the droplet contact angle and reduce the contact angle hysteresis [10]. The application of an electric field between the electrodes and a conductive liquid droplet attracts the liquid to the surface, decreasing the contact angle and increasing the surface wetting (Figure 1a,b). As a result, when the electrode directly beneath the droplet is grounded and a voltage is applied to the adjacent electrode, the contact angle of the droplet in the near energized electrode decreases, leading to asymmetric contact angles on two sides of the droplet, resulting in droplet motion towards the energized electrode [10]. Figure 1c,d show the actuation of a droplet placed on an EWOD device. The magnitude of contact angle change in EWOD depends on the applied voltage and is quantified by Young–Lippmann equation (please refer to the Supplementary Information). Though electrowetting has been historically demonstrated using bare, uncovered electrodes [11], EWOD is usually preferred because of two reasons. The first, is that the dielectric layer protects the liquid from the electrodes, allowing the application of high voltages, thereby resulting in stronger electrowetting force. The second, is that it reduces the contact angle hysteresis on the thin hydrophobic topcoat, making the droplet motion easier [12]. Not only do they increase electrowetting properties and facilitate droplet motion, the dielectric and hydrophobic layers also enable EWOD devices to be excellent platforms for biochemical assays, due to their inert and robust characteristics. This has been evident in all the examples of EWOD-DMF bioanalytical platforms. For instance, Srinivasan et al. developed an EWOD-based DMF device for colorimetric enzymatic glucose assays on serum, plasma, urine and saliva [13]. Hua et al. developed a DMF platform for automated, multiplexed, real-time polymerase chain reaction (PCR) assays [14]. Sista et al. developed a DMF platform for point-of-care detection of cardiac troponin 1, a common cardiac biomarker released during myocardial necrosis [15,16].

Furthermore, EWOD-DMF devices are particularly well suited for biochemical assays and potentially point-of-care (POC) testing applications. POC testing devices should be inexpensive, portable and robust to allow regular usage in any clinical setting [15]. EWOD-DMF devices are well apt for such applications as they allow direct electrical manipulation of fluids, without the need for direct contact with the electrodes or external, bulky mechanical pumps and valves, thus enabling a durable platform that can be easily integrated with cell phone analysis technology and developed into portable, handheld devices [15]. POC testing devices should also be capable of handling a wide range of laboratory tests and sample types [15]. EWOD-DMF devices offer the ability for repeatable use and flexibility to perform a wide range of experimental processes [15] across a unique landscape. Individual droplets can be controlled using a software program that allows easy modification of assay protocols without modification of the device itself [15]. Moreover, POC devices should be easily and economically mass produced. For traditional EWOD-DMF devices, low-cost and accessible fabrication remains a challenge. EWOD-DMF devices are usually manufactured on an insulative substrate (e.g., glass) in cleanroom facilities, through expensive, timely and complicated traditional microfabrication procedures such as optical lithography, etching and deposition processes [17]. This adds significant cost and complexity to these platforms hindering their large-scale production and applications for POC diagnostics. Clinical laboratories in many places, such as resource-poor settings and developing countries often have limited access to amenities and lack skilled technical personnel [18]. Moreover, specific diagnostic tests might not be available to a large amount of the population due to their high costs [18]. Statistics reported by the World Health Organization (WHO) suggest that over 3 million cases of tuberculosis (TB), which is one of the frequently recorded causes of death in Sub-Saharan Africa, remain undetected each year [18]. In 16 countries in Sub-Saharan Africa, about half the children born to HIV positive mothers are screened, and in some regions, less than 1 in 10 HIV infected children are tested [18]. These statistics portray the need for low-cost, easily accessible, POC diagnostic platforms to improve healthcare in low-income nations and countries. It is, therefore, important to consider limitations of infrastructure and cost while designing EWOD-DMF-based POC devices.

Considering the above requirements, we aimed to design and integrate a new class of low-cost EWOD-DMF devices, which are portable, easily prototype-able, rapidly integrate-able, and easily tailorable, which makes them accessible and appropriate for biochemical assays and potentially POC applications. To develop such devices, we used low-cost, scalable and easily accessible inkjet printing technology for the fabrication of EWOD-DMF platforms. Our platforms are made up of inexpensive, flexible inkjet-nanoparticle-printed (FINP) electronic apparatuses. By combining our FINP-DMF platform with a simple micro-controller circuitry and cell phone-based image analysis, along with a compact DC power source, we also demonstrated a portable platform with the capability of precise control, manipulation and analysis of samples. We used a smartphone camera to image the motion of droplets for offline analysis. However, a mobile app could also be developed for real-time analysis of images recorded from a smartphone to directly deliver results to end-users. Apart from being low-cost, portable and user friendly, our platform is also reusable and can be used to perform repetitive assays for different applications. Owing to its low-cost and easy fabrication method, our FINP-DMF platform has the potential to improve the POC diagnostics in resource-poor settings and low-income countries.

## 2. Materials and Methods

EWOD-DMF electrode array designs were drawn in Inkscape software, as shown in Figure 1f,g. Two types of electrode configurations were evaluated. The first, a square electrode configuration (electrode design A) and an interdigitated electrode configuration (electrode design B and C, respectively). The comparison of these two electrode configurations (square versus interdigitated) was pursued to determine the best geometry for maximum droplet speed and efficient manipulation. Electrode design A (Figure 1f) consists of an array of square-shaped electrodes (3 mm × 3 mm) separated by a gap of 200 μm between two electrodes. Electrode designs B and C (Figure 1g) are composed of an array of electrodes with a base size of 3 mm × 3 mm and a saw-tooth-like interdigitated design features 150 and 300 μm in width, respectively. FINP-DMF platforms were fabricated (Figure 1e) using silver nanoparticle ink, flexible microporous polyethylene terephthalate (PET) substrate and a commercial inkjet printer (Epson XP-440). The inkjet printer cartridges were filled with silver nanoparticle ink (Mitsubishi Paper Mills Limited) and inserted into the printer. The ink is a colloidal suspension of silver nanoparticles, isopropyl alcohol, ethylene glycol and ethanol. The average diameter of silver nanoparticles is ~20 nm. The thickness of the PET substrate is approximately 135 μm ± 12 μm. It was inserted in the paper slot of the printer, and the electrode designs were printed onto the substrate within minutes. The electrode arrays were then cut-out from the PET substrate using a scissor, and 1 μm Parylene C film was deposited onto the printed device using a Parylene coater. The parylene-coated device was then secured to a silicon wafer using double-sided tape and was placed in a spin coater. A hydrophobic layer of FluoroPel PFC-1101V (Cytonix, Beltsville, MD, USA) (1% Fluoropolymer solution) was spin-coated onto the device for 30 s at 1500 rpm. The device was then heated on a hot plate at 120 °C for 15 min. The use of the fluoropolymer solution reduces contact angle hysteresis allowing the droplet movement from one electrode to another. This fabrication procedure is low-cost, straight forward and rapid, compared to conventional cleanroom fabrication procedures. The minimum feature size of the electrodes obtained with this inkjet printing technique depends on the nozzle size of the printer. In our case, we can achieve a minimum feature size of 150 μm and a minimum gap of 125 μm between the two features. Figure 1e shows the schematic of the fabrication procedure of the FINP-DMF platform.

## 3. Results

### 3.1. Platform Characterization

Miniaturized platforms based on EWOD-DMF for biochemical assays should operate with fast droplet response times, minimum sample volumes, low power supply and a range of different sample solutions. These platforms require many considerations to generate a system that is dependable, inexpensive, easily manufacturable and generates accurate results in short periods. These considerations include proper electrode design and fabrication, suitable working parameters, the ability to handle a wide range of physiological relevant reagents and compositions. For this purpose, we showed the easy and cost-effective printing of conductive ink-based electrode arrays onto flexible substrates (Figure 1e). These inkjet-printed electrode patterns were generated within minutes using commercially available printers, eliminating the need for sophisticated equipment. Due to the importance of electrode design in droplet moving efficiency, two types of electrode configurations were tested. The first was a square electrode configuration (electrode design A) and the second was an interdigitated electrode configuration (electrode design B and electrode design C, respectively). Square and interdigitated electrode designs have been widely used in many existing EWOD studies [19,20]. Design A (Figure 1f) contains an array of square-shaped electrodes each 3 mm × 3 mm in size with a gap of 200 μm between two adjacent electrodes. The interdigitated electrode configuration (design B and C, Figure 1g) contains an array of electrodes with a base size of 3 mm × 3 mm with saw-tooth-like interdigitated features that are 150 μm in width for design B and 300 μm in width for design C. The detailed dimensions are shown in Figure 1g. The height of the electrodes was found to be approximately 300 nm. Additionally, the resistivity and conductivity of the electrodes was measured as approximately 1.6 × 10^−7^ Ω·m and approximately 6.25 × 10^6^ S/m, respectively. The sheet resistance was measured as approximately 0.5 Ω/sq. Here, we first performed initial characterization experiments to find the appropriate working parameters and electrode design (voltage, droplet volume, different fluids) for the rapid as well as effective manipulation and mixing of droplet suspensions followed by performing more clinically relevant experiments. Initial experiments were performed by optimizing the sample droplet volume. To move droplets continuously without any interruption on an array of electrodes, it is essential to use a droplet volume that not only covers the electrode directly beneath the droplet but also partially covers the adjacent electrodes. For the characterization of this work, we used three commonly used buffers in cell biological assays: DI water (buffer I), 1X phosphate-buffered saline (PBS, buffer II), and 5X PBS (buffer III). For these initial characterization experiments discussed within this section, buffer I was utilized. For all the experiments, droplets were placed on the FINP-DMF platform using laboratory pipettes. Droplet volumes of 20, 30 and 40 μL of buffer I were actuated within printed electrode arrays. It was found that 20 μL droplets failed to cover adjacent electrodes, interrupting their motion on the electrode arrays whereas 30 and 40 μL droplets were able to slightly overlap adjacent electrodes resulting in uninterrupted motion. To minimize the sample volume, which is one of the major requirements in most miniaturized platforms, we chose to work with 30 μL droplets for all the experiments described herein.

**Figure 2:**
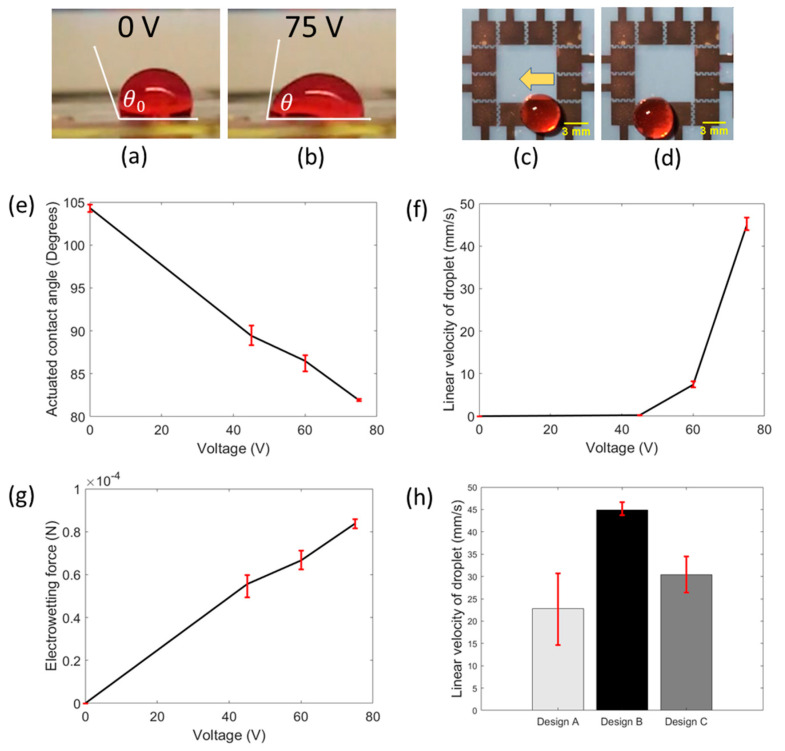
(**a**) Side view of the droplet with zero voltage as taken by a smartphone camera. The contact angle of the droplet is greater than 90° because of the underlying hydrophobic coating. (**b**) Side view of the droplet at an actuation voltage of 75 V as taken by a smartphone camera. The contact angle decreases due to the decrease in the solid–liquid surface tension. (**c**) Image showing a droplet placed on the FINP-DMF platform. (**d**) When the adjacent electrode is actuated, the droplet moves towards the actuated electrode. (**e**) Graph showing the variation of actuated contact angle with actuation voltage. (**f**) Graph showing variation of linear droplet velocity with the actuation voltage. (**g**) Graph showing the variation of calculated electrowetting force with the actuation voltage. (**h**) Bar graph showing the linear droplet velocity on electrode designs A, B and C.

In addition, for most EWOD-DMF devices (e.g., POC-based EWOD-DMF devices), it is essential to have a system that is both effective and efficient, one that can generate quick droplet motion with ideally minimum power consumption. To find the optimum driving force for droplet movement in our platform, five operating voltages were tested at 30, 45, 60, 75 and 90 V. At the lowest actuation voltage of 30 V, no droplet motion was observed, while at the highest voltage of 90 V, dielectric breakdown and hydrolysis was observed. However, proper actuation of droplets without dielectric breakdown or hydrolysis was achieved at 45, 60 and 75 V. The effect of actuation voltage on the droplet contact angle also plays an important role in droplet movement, as it has been shown that larger differences between actuated and non-actuated droplet contact angles generate larger electrowetting forces which yield a faster droplet movement [21]. According to the Young–Lippmann equation, the contact angle of the droplet upon actuation decreases with increasing actuation voltage. We experimentally investigated and verified this trend, as shown in Figure 2e. Droplet motion was recorded using a smartphone camera and its contact angle, upon actuation, was measured using imageJ, an open-source image analysis software. This experiment was repeated for three devices for each value of actuation voltage. According to our results, when no voltage was applied, the average contact angle of the droplet was 104.3° (non-actuated contact angle) and for 45 V, the average contact angle decreased to 89.4°. Similarly, on the application of 60 V and 75 V, the average contact angles were found to be 86.4° and 81.9°, respectively. The error bars shown in Figure 2e represent the maximum and minimum values of contact angle measurements obtained at each of the actuation voltages. In addition, it is important to maximize sample processing speed in our platform. We investigated the effect of different actuation voltages on the droplet velocity to find the proper voltage for the expeditious movement of droplets. Figure 2c,d show the movement of a droplet under the influence of actuation voltages. Three devices were tested. The measured average velocities of droplets were found to be 0.23, 7.4 and 44.9 mm/s at actuation voltages of 45, 60 and 75 V, respectively (Figure 2f). Error bars denote the maximum and minimum values of measured velocities at each actuation voltage. The resemblance of a parabolic curve (Figure 2f) was observed for the relationship between droplet velocity and actuation voltage, which agrees with previously reported work [21], where it was shown that the linear droplet velocity varies with the square of the actuation voltage [21]. Furthermore, the electrowetting force was calculated for all three functional actuation voltages (45, 60 and 75 V) as shown in Figure 2g. The electrowetting force was calculated to be greater with increasing actuation voltage, where at 45 V, the force was found to be 55 μN. At 60 V and 75 V, the force was found to be 66 μN and 84 μN, respectively. The increase in electrowetting force with respect to actuation voltage explains the increasing trend of linear droplet velocity. As the maximum droplet velocity and electrowetting driving force were observed at 75 V, this voltage was selected as the optimum value to carry out all the experiments and ensure rapid droplet response and faster biochemical assays. For all further experiments, we used an actuation voltage of 75 V.

Additionally, the high throughput capabilities of biochemical assays in EWOD-DMF devices is crucial for practical and clinical applications. It is important to appropriately consider electrode designs that produce controlled and rapid droplet movement so that numerous assay reactions and results can be attained quickly. Considering that, we initially studied the effect of different electrode configurations on linear droplet velocity. To determine the best geometry for maximum droplet speed and efficient manipulation, different high throughput electrode designs capable of providing high droplet velocity were designed and tested (A, B and C, Figure 1f,g). For these experiments, droplets were placed on one electrode and actuated to the neighboring electrode. The droplet motion was recorded using a smartphone camera. Using image analysis, the linear droplet velocity was extrapolated by dividing the distance that the droplet traveled (length of the electrode plus gap) by the time required for the droplet to move from one electrode to another. Figure 2h shows the bar plot of the linear droplet velocity of all three electrode designs. It shows that the droplet velocity was highest for design B (44.87 mm/s), followed by design C (30.43 mm/s), and lastly, design A (22.85 mm/s). For interdigitated electrode designs, such as B and C, it has been previously observed that linear droplet velocity is higher than that on the square electrodes. Our observations are consistent with this previously observed phenomenon [22]. A higher velocity on interdigitated electrode configurations (B and C) compared to square electrode configuration (A) is attributed to increased length of the contact line between the droplet and the actuated electrode, which in turn generates an increase in the driving force [22]. The difference between linear droplet velocities on design B and C can be explained by the fact that both designs have slightly different electrode designs (B has five and four fingers on opposing electrode pair; C has four and four fingers on opposing electrode pair) and thus, the increased length of the contact line of B, lends to a higher droplet velocity. As higher droplet velocities were observed on interdigitated electrode configurations (designs B and C), we chose to work with these designs for our further experiments.

**Figure 3:**
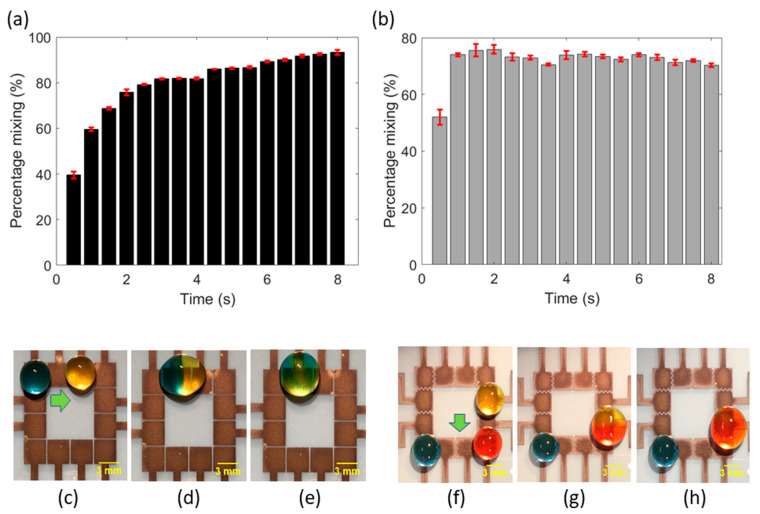
(**a**) Percentage mixing versus time for mixing of blue and yellow droplets as calculated from the relative mixing index. (**b**) Percentage mixing versus time for mixing of yellow and red droplets as calculated from the relative mixing index. The error bars indicate standard deviations. (**c**) Position of blue and yellow droplets at time (t) = 10.6 s on interdigitated electrode configuration. The initial volume of both the droplets is 30 μL. (**d**) The blue droplet moves towards the yellow droplet and starts merging with it (t = 10.7 s). (**e**) Mixing of blue and yellow droplet yields a green-colored droplet (t = 18 s). (**f**) Position of yellow and red droplets at t = 5.4 s on square electrode configuration. The initial volume of both the droplets is 30 μL. (**g**) Yellow droplet moves towards the red droplet and starts merging with it (t = 5.7 s). (**h**) Yellow and red droplets mix with each other to yield a single droplet (t = 13 s).

Additionally, for biochemical assays performed on EWOD-DMF devices, droplets of different reagents are often dispensed from different sites on the device, moved towards each other, and made to coalesce to mix both the reagents. For example, Ng et al. reported a proof-of-concept EWOD-DMF platform for performing a rubella virus (RV) IgG immunoassay [23]. RV IgG antibodies were detected using an indirect ELISA involving several droplets mixing steps to capture the IgG antibody and incorporate chemiluminescent labels to the sample. Droplet mixing is thus a fundamental operation that needs to be performed on EWOD-DMF devices to make them suitable for point-of-care biochemical assays and diagnostic tests, for instance. To characterize the mixing of two droplets on our FINP-DMF platform, we performed proof-of-concept experiments with droplets colored with different dyes and mixed them using the FINP-DMF platform, where the distinct color droplets served as models for different reagents. We initially investigated the effect of electrode geometry on droplet mixing by performing mixing experiments with the square electrode configuration (design A, shown in Figure 1f) and an interdigitated electrode configuration (design C, shown in Figure 1g). Figure 3c–e show the mixing of blue and yellow droplets on a square electrode configuration (design A), and Figure 3f–h show the mixing of red and yellow droplets on the interdigitated electrode configuration (design C), under the actuation of 75 V. Moreover, the level and rate of mixing achieved is of equal importance in biochemical assays. EWOD-DMF devices must ensure that two droplets mix completely and homogeneously in short periods. Mixing of droplets is dominated by diffusion because of their laminar state, which is a slow process. It is therefore necessary to quantify the level and rate of mixing to meet the assay design objectives of complete reaction mixing rapidly. To characterize the rate of mixing in our FINP-DMF platform, we calculated the relative mixing index previously described in the literature [21] for both design A and C. We performed image analysis using MATLAB and calculated the percentage of mixing (level of mixing) using the mathematical formula for RMI (defined in Supplementary Information). A 12-megapixel smartphone digital camera at a rate of 30 frames/s and a tripod holder was used to fix the camera in place. A 150 W halogen variable intensity fiber optic light source was used for illuminating the device. Figure 3a shows the graph of mixing percentage versus time for design A (Figure 3c–e). It can be observed that mixing percentage rapidly increased with time within the first one or two seconds and remained almost constant thereafter. The rate of mixing at any given time can be found by calculating the slope of the curve at that time point. The maximum slope was observed between 0 s to 1 s, and the rate of mixing (slope) within this time interval was found to be 60%/s. A similar trend was observed for mixing on electrode design C (Figure 3f–h), where the percentage mixing increased rapidly within the first second and then remained almost constant for the rest of the time duration (Figure 3b). The maximum slope (rate of mixing) was observed in 0 s to 1 s and was calculated to be 78%/s. The higher slope of the curve (rate of mixing) for design C, compared to design A, may be attributed to higher linear droplet velocity on interdigitated electrode configuration, which enhances the natural oscillations of the droplet during its coalescence, resulting in rapid diffusion and mixing. To improve the mixing percentage, other methods such as moving the droplet rapidly back and forth or agitating the droplet with acoustic waves, could be employed.

**Figure 4:**
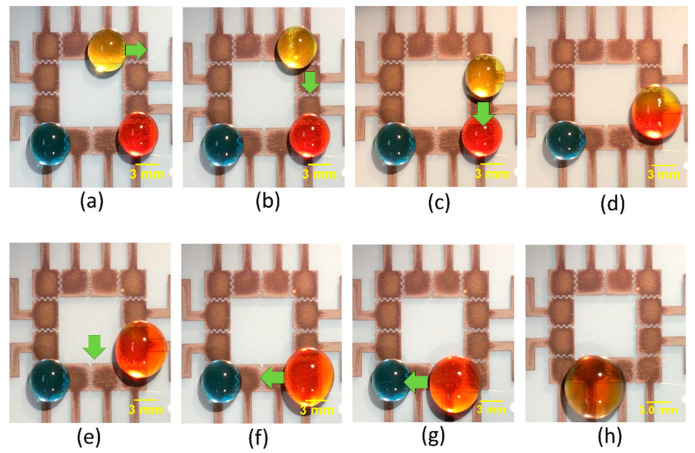
Sequence of images showing the motion and mixing of droplets on the FINP-DMF platform with interdigitated electrode design (design C). (**a**) Position of yellow, red and blue droplets at time (t) = 3.6 s. Initial volume of all droplets is 30 μL. (**b**) Position of droplets at t = 3.7 s. (**c**) Position of droplets at t = 4.9 s. (**d**) The yellow droplet moves towards the red droplet and starts merging with it (t = 5.7 s). (**e**) Yellow and red droplets mix with each other to yield a single droplet (t = 23.9 s). (**f**) The yellow–red droplet mixture then moves towards the adjacent electrode (t = 24.2 s). (**g**) Position of the blue droplet and yellow–red mixture droplet at t = 27.6 s. (**h**) The yellow–red droplet mixture moves towards the blue droplet and mixes with it (t = 37.1 s).

Having studied the effect of different parameters on moving and mixing of droplets, we moved on to demonstrate the utility of our platform to perform integrated droplet movement and sequential mixing operations. A 30 μL yellow droplet was initially placed on the top right corner, and a blue and a red-colored droplet were placed on the bottom left and right corner, respectively. While keeping the red droplet stationary, the yellow droplet was manipulated by applying the actuation voltage to the adjacent upper electrodes and grounding the electrode on which the droplet currently resides. By sequentially applying voltages in this way, we could move the yellow droplet. The yellow droplet was moved from the top right corner towards the bottom right corner, where it was mixed with the red colored droplet. This resultant droplet mixture was then moved towards the bottom left corner, where it was mixed with a blue-colored droplet. Figure 4a–h show time sequence images of simultaneous movement and mixing using design C of the FINP-DMF platform. These experiments demonstrated the capability of our FINP-DMF platform to manipulate multiple droplets and perform different mixing and movement operations, which is often required in digital biochemical assays.

### 3.2. Platform Characterization for Clinically Relevant Reagents

**Figure 5:**
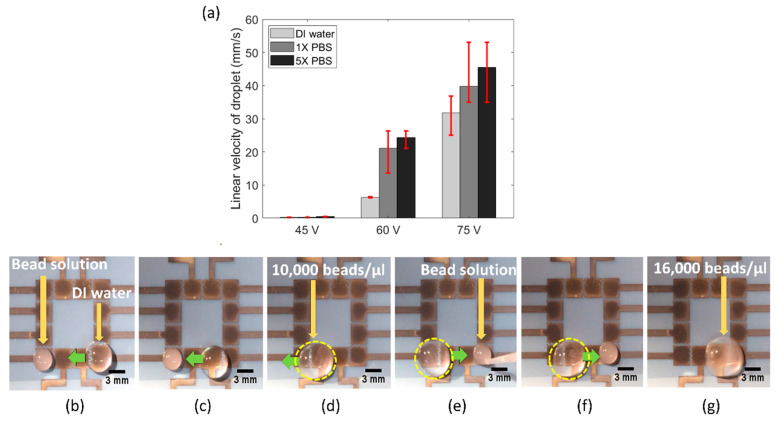
(**a**) Graph showing a variation of linear droplet velocity at different actuation voltages for buffer I, buffer II and buffer III droplets. (**b**) Position of concentrated bead solution droplet (10 μL) and buffer I droplet (30 μL) at time (t) = 9 s. (**c**) Buffer I moves towards the adjacent electrode (t = 12 s). (**d**) Buffer I moves towards the bead solution and mixes with it (t = 18 s). (**e**) A 10 μL bead solution droplet is again placed on the rightmost electrode (t = 24 s). (**f**) Mixture of bead solution and buffer I moves towards the right electrode (t = 28 s). (**g**) The droplet mixture mixes with the new bead droplet on the rightmost electrode (t = 36 s).

Next, we tested the functionality of our FINP-DMF platform to work with pseudo biospecimen regents. Several biochemical assays are performed with a range of conductive media, such as phosphate saline buffer, lysis buffer and ethylene diamine tetra acetic acid (EDTA). As a result, EWOD-DMF devices utilized in biochemical assays for POC applications must be robust and operate with a range of reagents. To demonstrate the utility of our platform with such physiological conductive buffers, we performed experiments with a variety of liquids commonly used in biochemical assays, including buffer I (DI water), buffer II (1X PBS) and buffer III (5X PBS) solutions. Specifically, we quantified the linear droplet velocity of both these buffers at different actuation voltages, with the main aim of investigating the effect of buffer conductivity on droplet velocity. Figure 5a shows the bar plot of linear droplet velocity of buffer I (conductivity approximately 0.055 μS/cm), buffer II (conductivity approximately 15 mS/cm) and buffer III (conductivity approximately 75 mS/cm), at three different actuation voltages. It was observed that at any given actuation voltage, the linear droplet velocity was highest for buffer III, followed by buffer II and buffer I. It has been previously observed that the electrowetting force increases with liquid conductivity [24]. Though the physics is still not well developed, it is theorized that higher conductivity liquids provide more charge to be produced at the solid–liquid interface and results in reduced surface tension. This is likely the reason why we observed the highest droplet velocity for buffer III and lowest droplet velocity for buffer I.

Biochemical assays often require manipulation, mixing and dilution of biological specimen samples, such as cell or DNA samples diluted with lysing buffer. Hence, we also examined the utility of our FINP-DMF device to manipulate and mix pseudo biospecimen suspensions and reagent and generate diluted suspensions. To do this, we manipulated a concentrated solution of 10 μm polystyrene (PS) beads (SPI supplies, USA) sample, where beads were used as models of cells. We placed a 10 μL droplet of a concentrated (40,000 beads/μL) PS beads solution on the leftmost electrode and a 30 μL water droplet on the rightmost electrode on our FINP-DMF platform (Figure 5b). We then moved the droplet towards the bead solution and mixed it to generate a diluted bead droplet with a concentration of 10,000 beads/μL (Figure 5c,d). We placed a second 10 μL concentrated bead droplet (40,000 beads/μL) on the rightmost electrode (Figure 5e) and moved the previously generated diluted bead droplet towards the concentrated bead droplet where it was mixed. This resulted in a droplet with a new concentration of 16,000 beads μL (Figure 5f,g). These results demonstrated the utility of our FINP-DMF platform to move and mix droplets of different content concentrations. Thus, our FINP-DMF device could potentially be useful for performing various kinds of sample preparation and dilution steps for diagnostic tests.

### 3.3. Portable FINP-DMF Platform

**Figure 6:**
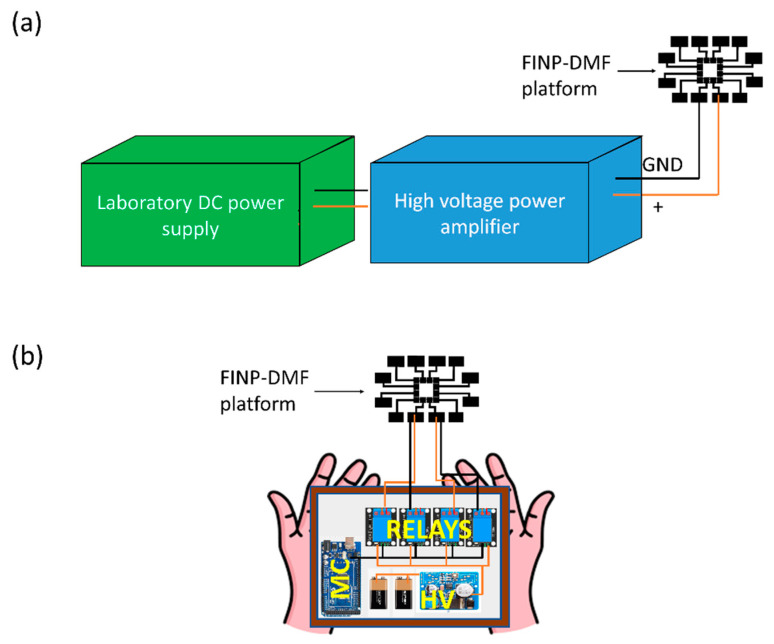
(**a**) Schematic of a bulky control circuitry, a laboratory DC power supply along with a high voltage amplifier. (**b**) Schematic of the portable control circuitry. Two 9 V batteries in series are used with the miniaturized high voltage, step-up DC–DC converter (HV), along with the microcontroller (MC), and relay circuitry to create a lightweight and compact version of control circuitry for switching electrodes on and off.

EWOD-DMF devices adapted for applications such as POC testing should be composed of designs that are miniaturized, affordable, portable and user-friendly for rapid and thorough assay-processing performance on a single device. For the experiments so far, the commercially available experimental setup, consisting of a laboratory DC power supply and high voltage amplifier (Figure 6a), were used. However, this type of control system has large dimensions and weight. For example, the laboratory DC power supply and high voltage amplifier would fit in a box of dimensions 10 inches × 10 inches × 6 inches and would weigh 11 lbs. For most applications, a much smaller and lighter system is desired where the platform can be easily handled and transported in resource-limited settings. To miniaturize and automate our FINP-DMF platform, we developed a portable, automated and lightweight control system for the manipulation of droplets. The portable platform consists of two 9 V alkaline batteries, a miniaturized step-up DC–DC converter (NCH6100HV high voltage DC power supply), an Arduino ATMEGA 2560 microcontroller and Arduino-compatible relay modules (switching time: 5 ms) for precisely switching electrodes on/off. The step-up DC–DC converter needs a DC input ranging from 12–24 V and provides a DC output in the range 85–235 V. The series combination of two 9 V batteries supply 18 V as input to the DC–DC converter, which in turn provides an output of approximately 127 V. To avoid any damage to our devices during experiments, the voltage was lowered to 75 V using a voltage divider circuit constructed from an appropriate combination of resistors. Arduino IDE software was used to program the switching circuit. The relays were switched “on” for 3 s so that the droplet moved from one electrode to another and then switched “off.” After a delay of 500 ms, the relays were again switched “on” to move the droplet over the electrodes in a sequence. Figure 6b shows the schematic of the portable circuit, which is a miniaturized and compact system compared to the bulky set-up shown in Figure 6a, and fits in a smaller 7 inches × 3 inches × 3 inches box and weighs about 2 lbs. Preliminary experiments with our battery-powered portable circuit have shown that we can successfully manipulate buffer I droplets of 30 μL at a driving voltage of 75 V. By reducing the size FINP-DMF system, we have demonstrated that we can successfully automate the process of manipulating droplets and easily program the platform to potentially perform a wide variety of assays.

## 4. Discussion

Since the discovery of the EWOD [25], several research groups have fabricated EWOD-based DMF platforms for a variety of applications such as dried blood spot (DBS) analysis [26], single-molecule detection [27] and microRNA analysis. However, the majority of the platforms reported in the literature are manufactured using expensive cleanroom processes [28,29,30]. For example, the DMF platform used for DBS analysis was fabricated in a cleanroom using expensive processes like photolithography and etching [26]. However, it is extremely difficult to establish such facilities in developing countries. According to a recent study, the estimated capital per square foot of the cleanroom could range from US $1800–4000, including the cost of the cleanroom building structure and support systems [18]. It generally costs about US $2000 per square foot for building a class-10 cleanroom and incurs an operating cost of US $1 million [18]. These numbers show how expensive and difficult it is to set up and operate a cleanroom and emphasize the need for cleanroom-free manufacturing methods for DMF devices to enable their widespread use. Our FINP-DMF platforms could potentially provide a cleanroom-free solution for manufacturing low-cost DMF platforms for various biochemical assays and POC diagnostics. Using just a commercial office inkjet-printer, we can print electrode patterns for hundreds of DMF devices within minutes. Our method can not only potentially reduce the cost and increase manufacturing throughput, but can also make these devices easier accessible to others. Clinical or research laboratories in developing countries often lack essential facilities. Using a commercial office inkjet printer, which is generally available everywhere, FINP-DMF platforms can be quickly prototyped. Due to the minimal need for fabrication resources, such platforms can potentially be prototyped in schools or educational institutions to teach students and encourage them to innovate. Apart from the fabrication of FINP-DMF platforms, our low-cost inkjet-printed electrode fabrication method could also be utilized for developing other types of miniaturized devices such as single-cell impedance cytometry platforms [31] and dielectrophoretic cell separation devices [32].

For many applications, including biochemical assays for point-of-care applications, it is necessary to develop more portable and compact control circuitry considering the limitations in space, funding and cost. To reduce the cost and increase the portability of the platform, we transitioned to a portable, lightweight and low-cost control circuit utilizing two 9 V batteries along with a miniaturized step-up DC–DC converter that supplies the actuation voltage, in combination with Arduino and relays. The use of microcontroller and the Arduino IDE software allows for programming the droplet motions on the platform and thus can be used to develop pre-programmed assays. This functionality reduces the dependency on skilled technicians, as the programmed platform can perform assays, and directly deliver the results to the end-users. It is worth noting that even though we used a computer to program the microcontroller, a similar smartphone-based application for controlling the FINP-DMF platform could also be developed to make the system more compact. Furthermore, using powerful cloud computing, such a smartphone-based application results could be accessible to clinicians. This would particularly benefit patients in low-resource settings, where access to clinicians is limited, as they can consult a clinician in a remote city by directly sharing the assay results over the cloud. In this study, we recorded droplet motion with a smartphone camera for offline analysis. However, a smartphone application for real-time image analysis, particularly for assays like ELISA could potentially be developed.

Actuation voltages in EWOD devices depend on the dielectric constant of the insulative layer as well as its thickness. We used a 1 μm thick Parylene C insulative layer and achieved the optimum actuation voltage of 75 V. However, it may be possible to lower the actuation voltage by using another insulating material of lower thickness and higher dielectric constant (e.g., barium strontium titanate (BST)) [33].

Previously, some research groups have reported inkjet-printed DMF platforms for other applications. Dixon et al. reported an inkjet-printed, roll-coated DMF platform for performing miniaturized diagnostic assays [17]. Although the performance of their device was on par with conventional DMF devices, one major drawback with their technique is that it takes more than one day to complete fabrication as the inkjet-printed electrodes need to be dried overnight after printing and before coating the dielectric and hydrophobic layer [17]. Compared to their approach, our inkjet printing technique is rapid (approximately 5 min), and the electrodes can be coated with the dielectric and hydrophobic layer right after printing. This saves time and enables the manufacturing of a large number of FINP-DMF devices in a very short time. Another study reported inkjet-printed DMF devices, however, they used an industrial-grade printer to fabricate their platforms. Compared to their DMF platforms, the FINP-DMF platforms are much easier to fabricate as they can be batch-manufactured with a simple office printer, which is easily accessible and low-cost compared to an industrial-grade printer. Considering the low-cost, ease of manufacturing, portability and the capability of using it with a smartphone, we envision our FINP-DMF platform to have a potential impact in the field of DMF-based POC diagnostics.

## 5. Conclusions

In conclusion, we have reported an inexpensive, scalable and easily manufactured FINP-DMF platform and have demonstrated its utility to move and mix droplets in open EWOD configuration. These platforms can also be redesigned to operate in a closed EWOD configuration and could be optimized for other functionalities such as droplet splitting and dispensing. Due to its rapid, inexpensive and straightforward fabrication process compared to conventional microfabrication procedures, as well as the integration of low-cost and simple control circuitry, our platforms could potentially have applications in biochemical assays for POC applications. For example, infectious diseases such as tuberculosis are quite common in low-income countries. Polymerase chain reaction (PCR) is one of the methods for the diagnosis of such infectious diseases and involves a process of heating and cooling the sample at various temperatures. PCR is usually performed in several processing machines, which are often expensive and unsuitable for use in extremely low-resource areas. An integrated, miniaturized, inexpensive and on-chip PCR diagnostic kit can potentially be developed by employing FINP-DMF to shuttle sample droplets between different temperature zones and perform PCR. However, one should also note that before any device can be used in clinical settings for POC applications, it must pass all stages of the FDA approval process. We envision our FINP-DMF platform to have a potential impact in biochemical assays for POC diagnostics, one which could potentially make a significant impact in low-income countries’ current health practices, provided all FDA regulations are met.

## Supplementary Materials

The following are available online at https://www.mdpi.com/1424-8220/20/12/3593/s1, Expanded theory of electrowetting-on-dielectric is provided in the supplementary information section.
