# Supplementary material for: A Low-Cost, Disposable and Portable Inkjet-Printed Biochip for the Developing World
Source: Sensors (Basel). 2020 Jun 25;20(12):3593. doi: 10.3390/s20123593 (PMC7348740; doi:10.3390/s20123593)
Supplement: Supplementary file 1 [file sensors-20-03593-s001.pdf]

Supplementary Information

1. Theory

When an electric potential is applied between a metal-electrolyte interface, charges are redistributed and accumulate at the solid-liquid interface, forming an electrical double layer (EDL), which changes the surface energy of the solid-liquid interface [1]. Repulsion of like charges at the solid-liquid interface reduces the work required to expand the surface area, leading to a decrease in the associated surface tension and induces a change in the contact angle [1]. This phenomenon is called electrowetting [2]. The relationship between contact angle and applied electric potential is described using the Lippmann-Young equation, which can be derived using different approaches – the classical thermodynamic approach, the energy minimization approach, and the electromechanical approach. Here we present the classical thermodynamic approach in detail. The other two approaches can be found elsewhere [3].

a. The classical Thermodynamic Approach

Consider a droplet of conductive liquid (electrolyte) placed on a smooth metal surface. The metal surface is in direct contact with the electrolyte, and a small potential difference is applied such that no current flows through the liquid and no Faradaic reactions take place on the metal surface [3]. Due to the application of an electric field, an electrical double layer (EDL) is formed in the liquid which is in contact with the metal surface [3]. The mathematical relationship between the surface charge distribution, surface tension and electric potential can be derived from Gibb's interfacial thermodynamics [3], which yields:

$$-\rho_{SL} = \frac{d\gamma_{SL}^{eff}}{dV} \quad (1)$$

Here γ_{SL}^{eff} is the effective surface tension of the solid-liquid interface, ρ_{SL} is the electric-field induced surface charge density at the solid-liquid interface, and V is the electric potential [3]. Assuming that all the ions in the EDL are located at a fixed distance d_H (which is usually in the order of few nanometers) from the surface, the EDL can be considered to have a fixed capacitance (Helmholtz layer capacitance) per unit area[3] and can be expressed as:

$$C_H = \frac{\epsilon_0 \epsilon_l}{d_H} \quad (2)$$

where $\boldsymbol{\epsilon_0}$ is the vacuum permittivity, ϵ_l is the relative permittivity of liquid. Integration of equation 1 yields:

$$\gamma_{SL}^{eff}(V) = \gamma_{SL} - \int_{V_{pzc}}^{V} \rho_{SL} dV \quad (3)$$

The mathematical relationship between C_H, ρ_{SL}, and V is given by equation 4 [3]:

$$C_H = \frac{d\rho_{SL}}{dV} \quad (4)$$

Integrating equation 4 and combining it with equation 3 yields:

$$\gamma_{SL}^{eff}(V) = \gamma_{SL} - \int_{V_{pzc}}^{V} C_H V dV = \gamma_{SL} - \frac{C_H}{2}\left(V - V_{pzc}\right)^2 \quad (5)$$

Here, V_{pzc} is the potential at zero charge. When a solid is immersed in an electrolyte at zero voltage, spontaneous charges appear at the solid surface, and V_{pzc} represents the voltage needed to compensate this process [3]. V in equation 5 is the voltage of the electrode relative to a reference electrode. Here we have assumed that most of the voltage drop occurs across the EDL, and there are no ohmic losses in the liquid [3]. We now apply Young's equation at zero potential and potential V, which yields:

$$\gamma_{SG} - \gamma_{SL} = \gamma_{LG} \cos\theta_0 \quad (6)$$

$$\gamma_{SG} - \gamma_{SL}^{eff}(V) = \gamma_{LG} \cos\theta \quad (7)$$

and θ_0 are the contact angles after actuation and before actuation, respectively. Subtracting equation 6 and 7 and then substituting equation 5 yields the Lippmann-young equation:

$$\cos\theta = \cos\theta_0 + \frac{C_H}{2\gamma_{LG}}\left(V - V_{pzc}\right)^2 \quad (8)$$

In an EWOD configuration, the EDL forms at the surface of the dielectric layer[3]. We can thus consider EDL to be in series with the dielectric layer [3]. Thus, C_D can be written as:

$$C_D = \frac{\epsilon_0 \epsilon_D}{d} \quad (9)$$

Where ϵ_d is the relative permittivity of the dielectric layer, and d is the thickness of the dielectric layer. Since d_H (usually in the order of few nanometers) << d (usually in micrometers) and $\epsilon_D < \epsilon_l$, $C_D << C_H$. The equivalent capacitance of the system can be given by the relation:

$$C_{eq} = (C_D * C_H)/(C_D + C_H) \quad (10)$$

can be approximated to $C_{eq} = C_D$. Thus, in an EWOD configuration, the voltage drop occurs in the dielectric layer, and the voltage drop across EDL can be neglected [3]. Accordingly, equation 5 can be modified as:

$$\gamma_{SL}^{eff}(V) = \gamma_{SL} - \frac{C_D}{2}V^2 = \gamma_{SL} - \frac{\epsilon_0 \epsilon_D}{2d}V^2 \quad (11)$$

Here we have assumed that the dielectric layer does not give rise to spontaneous electric charge at the surface and thus, we have neglected V_{pzc} [3]. By combining Young's equation with equation 11, we obtain the Lippmann-young equation for EWOD:

$$\cos\theta = \cos\theta_0 + \frac{C_D}{2\gamma_{LG}}V^2 \quad (12)$$

The term $\frac{C_D}{2\gamma_{LG}}V^2$ is called the electrowetting number (η).

Electrowetting Force and Droplet Motion on EWOD Platform

Chaudhury et al. and Subramanian et al. have previously shown that droplets can be moved on a surface using a difference or gradient in wettability [4,5]. When a droplet is located on the boundary between lyophobic and lyophilic regions, the droplet moves to the lyophilic region, if the contact angle hysteresis is small. The EWOD principle can be used to move droplets in a similar way. The electrodes are covered with a dielectric layer and a hydrophobic topcoat to reduce the contact angle hysteresis. When the droplet is located on the boundary between the actuated and non-actuated electrode, the electrowetting force is applied on the contact line located above the actuated electrode, and the capillary

force is exerted on the contact line located on the lyophobic region [3] . The resultant force is located towards the actuated electrode and if it is sufficient to overcome the contact angle hysteresis, the droplet moves towards the actuated electrode [3]. The electrowetting force is because of the Maxwell stress tensor and can be translated as a capillary effect using Lippmann-Young equation. The electrowetting force during the motion of droplet from one electrode to another remains constant and is given by:

$$F_{e,motion} = e\gamma_{LG}(cos\theta_a - cos\theta_{na}) \quad (13)$$

Where e is the width of the electrode, θ_a and θ_{na} are actuated and non-actuated contact angles ,respectively. The derivation of this equation can be found elsewhere [6].

EWOD platform consists of a base plate with an array of discrete electrodes used to actuate sessile droplets. Such platforms are often referred to as open EWOD platforms and are usually preferred for mixing operations because natural oscillations during droplet coalition enhances the mixing process [3,7]. In the present work, we have designed an open EWOD platform with our FINP electronic apparatus and demonstrated its utility for moving and mixing droplets.

Calculation of Mixing Rate Using Relative Mixing Index

The mixing rate of two colored liquids can be calculated by a variety of image analysis methods. Several mathematical indices, which can be calculated by image analysis, have been developed in order to quantify the extent of mixing [7]. Among various mixing indices developed by the research community, relative mixing index (RMI) is capable of providing the most accurate estimate of mixing because of its insensitivity to light intensities and span of intensity distributions [7]. Thus, we chose to use RMI to quantify the mixing of droplets on our FINP-DMF platform. The mathematical formula for quantifying RMI can be defined as:

$$RMI = 1 - \frac{\sqrt{\frac{1}{N}\sum_{i=1}^{N}(I_i - I_i^{perf\ mix})^2}}{\sqrt{\frac{1}{N}\sum_{i=1}^{N}(I_i^0 - I_i^{perf\ mix})^2}} \quad (14)$$

Where N is the total number of pixels, I_i is the intensity at pixels i, I_i^0 is the intensity at pixel I if no mixing or diffusion occurs, $I_i^{perf\ mix}$ is the intensity of perfectly mixed solution at pixel i. The percentage of mixing can be found by multiplying RMI by 100% (% mixing = RMI x 100%).

2. References

1. Moon, H.; Cho, S.K.; Garrell, R.L.; Kim, C.J. Low voltage electrowetting-on-dielectric. *J. Appl. Phys.* **2002**, *92*, 4080–4087, doi:10.1063/1.1504171.
2. Shen, H.H.; Fan, S.K.; Kim, C.J.; Yao, D.J. EWOD microfluidic systems for biomedical applications. *Microfluid. Nanofluidics* **2014**, *16*, 965–987, doi:10.1007/s10404-014-1386-y.
3. Berthier, J. *Electrowetting Theory*; 2013; ISBN 9781455725502.
4. Mk, C.; GM, W. How to make water run uphill. *Science (80-.).* **1992**, *256*, 1539–1541.
5. Subramanian, R.S.; Moumen, N.; McLaughlin, J.B. Motion of a Drop on a Solid Surface Due to a Wettability Gradient. *Langmuir* **2005**, *21*, 11844–11849, doi:10.1021/la051943i.

6. Berthier, J.; Dubois, P.; Clementz, P.; Claustre, P.; Peponnet, C.; Fouillet, Y. Actuation potentials and capillary forces in electrowetting based microsystems. *Sensors Actuators, A Phys.* **2007**, *134*, 471–479, doi:10.1016/j.sna.2006.04.050.
7. Hashmi, A.; Xu, J. On the Quantification of Mixing in Microfluidics. *J. Lab. Autom.* **2014**, *19*, 488–491, doi:10.1177/2211068214540156.
